# Environmental Quality Assessment of Bizerte Lagoon (Tunisia) Using Living Foraminifera Assemblages and a Multiproxy Approach

**DOI:** 10.1371/journal.pone.0137250

**Published:** 2015-09-15

**Authors:** Maria Virgínia Alves Martins, Noureddine Zaaboub, Lotfi Aleya, Fabrizio Frontalini, Egberto Pereira, Paulo Miranda, Miguel Mane, Fernando Rocha, Lazaro Laut, Monia El Bour

**Affiliations:** 1 Universidade do Estado do Rio de Janeiro—UERJ, Faculdade de Geologia, Av. São Francisco Xavier, 524, Maracanã. 20550–013 Rio de Janeiro, RJ, Brasil; 2 Universidade do Estado do Amazonas–UEA, Av. Djalma Batista, 3578, Flores, CEP 69050–010, Manaus, Brasil; 3 Universidade de Aveiro, Dpto. Geociências, Campus de Santiago, 3810–193, Aveiro, Portugal; 4 Institut National des Sciences et Technologies de la Mer, 1934–2025 Salammbô, Tunisia; 5 Université de Bourgogne Franche-Comté, Laboratoire de Chrono-Environnement, UMR CNRS 6249, Place Leclerc, F-25030 Besançon Cedex, France; 6 Università degli Studi di Urbino "Carlo Bo", Dipartimento di Scienze della Terra, della Vita e dell'Ambiente, Campus Scientifico Enrico Mattei, Località Crocicchia, 61029 Urbino, Italy; 7 Laboratório de Micropaleontologia–LabMicro, Universidade Federal do Estado do Rio de Janeiro–UNIRIO, Av. Pasteur, 296—Urca—Cep 22290–240, Rio de Janeiro, Brazil; Institute of Tibetan Plateau Research, CHINA

## Abstract

This study investigated the environmental quality of the Bizerte Lagoon (Tunisia) through an integrated approach that combined environmental, biogeochemical, and living benthic foraminiferal analyses. Specifically, we analyzed the physicochemical parameters of the water and sediment. The textural, mineralogical, and geochemical characteristics of the sediment, including total organic carbon, total nitrogen, simultaneously extracted metals (SEM), acid volatile sulfides (AVS), chlorophyll *a*, CaCO_3_, and changes in bacterial populations and carbon isotopes were measured. The SEM/AVS values indicated the presence of relatively high concentrations of toxic metals in only some areas. Foraminiferal assemblages were dominated by species such as *A*. *parkinsoniana* (20–91%), *Bolivina striatula* (<40%), *Hopkinsina atlantica* (<17%), and *Bolivina ordinaria* (<15%) that cannot be considered typical of impacted coastal lagoons both in Mediterranean and northeast Atlantic regions. The results of this work suggest that Bizerte Lagoon is a unique setting. This lagoon is populated by typical marine species that invaded this ecosystem, attracted not only by the prevailing favorable environmental conditions but also by the abundance and quality of food. The results indicate that the metal pollution found in some areas have a negative impact on the assemblages of foraminifera. At present, however, this negative impact is not highly alarming.

## Introduction

The European Water Framework Directive (WFD, Directive 2000/60/EC) establishes that the ecological status of water bodies is assessed in terms of the quality of their biological, physicochemical, and hydro-morphological elements. Such criteria are well defined for coastal waters, while they are still under discussion for transitional waters, such as coastal lagoons. Coastal lagoons and estuaries, which generally support high levels of biological productivity, represent a continuum between continental and marine aquatic ecosystems, and they are commonly characterized by a shallow water depth, strong fluctuations of physicochemical parameters, and marked gradients, such as in salinity [1], organic matter quantity and quality, oxygen availability, and the occurrence of pollutants [2].

Lagoons are subjected to significant environmental changes due to both natural and anthropogenic influences. These influences include engineering constructions that alter their natural circulation patterns, the extraction or deposition of mineralogical materials, the exploitation of biological products, and water and sediment pollution that are the main causes of environmental degradation [2], [3], [4]. Different proxies, such as biogeochemical indicators [2], [5], [6], [7], [8], have been used to assess the water and sediment quality of coastal lagoons and to evaluate their environmental vulnerability.

Among these, benthic foraminifera are one the largest applied and most effective bioindicators of environmental quality and, therefore they have been used to better understand changes in the physicochemical parameters of marine and transitional environments [7], [9], [10], [11]. Benthic foraminifera occur in many environments, including transitional settings such as saltmarshes, river estuaries, coastal lagoons and bays as well as oceanic basins [12]. Their distribution is controlled by many factors, such as temperature, salinity, oxygen, and sediment grain size or substrate type [13], and changes in the types and amounts of food [12], [14], [15], [16]. Some species colonize oxic sediment-water interfaces, while others tolerate oxygen deficiency and episodic anoxic conditions [17], [18]. Benthic foraminiferal assemblages are prevalently influenced by sediment characteristics but they are also affected by sediment pollution [2], [8], [19], [20], [21], [22], [23].

Other elements, such as mineralogical, physicochemical, geochemical and biological proxies, should be considered and integrated to assess lagoonal ecological quality [2], [23], as also suggested by the WFD. Among these, the available concentrations of heavy metals should be analyzed in sediments and/or waters to investigate the extent of the impact of anthropogenic activities, as well as to evaluate their hazard level. Sediments generally retain pollutants longer than water, which is, in large part, renewed by tides in coastal systems [24], [25], [26], [27]. Relatively high available concentrations of heavy metals adsorbed to sediments may be toxic to benthic organisms [24], [25], [26]. Moreover, pollutants deposited in sediments can be resuspended into the water column and affect benthic and planktonic habitats [28], [29]. Most of the work based on foraminiferal responses to metal pollution is based on total metal concentrations in the sediments, yet very little is known about the responses of living benthic foraminifera to relatively high concentrations of available metals in sediments, whose levels are of concern for biota [2], [30].

This work aims to study the living benthic foraminiferal assemblages of the Bizerte Lagoon, as well as the possible factors driving their distribution. This study is the first to evaluate the sustainability and the sediment quality of Bizerte Lagoon using biogeochemical proxies, including living benthic foraminifera, bacteria as well as the first to predict how coastal ecosystems are responding to the combined effects of eutrophication and pollution pressure. In the broader context of the study of coastal lagoons and other transitional environments, this work advances our knowledge because it shows that: the analysis of sediment quality based only on pollutant assessment can result in an inconclusive knowledge about the negative impact of contaminants on living organisms.

## Study Area

**Fig 1:**
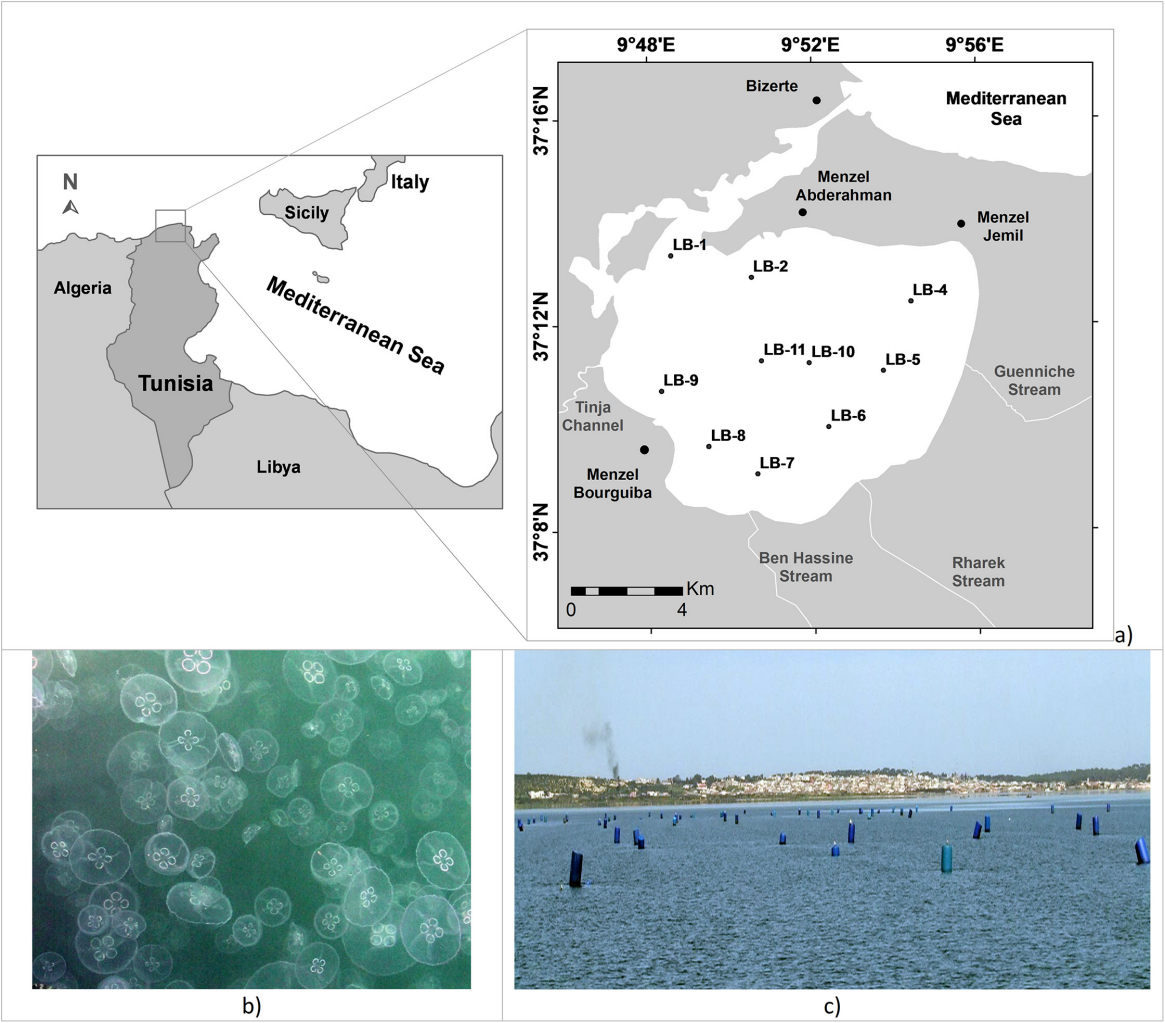
a) Study area in the Bizerte Lagoon (adapted from OTC, 1992, Map of Bizerte Lagoon office de la topographie et de la cartographie de Tunisie). The studied stations are labeled with numbers. The main towns and streams are indicated. b) Photo of the surface water column showing the cnidarian bloom. c) Photo showing suspended structures used in mollusks culture.

The Bizerte Lagoon is a Mediterranean transitional ecosystem located in north Tunisia (latitude: 37°8’–37°14’N, longitude 9°46’–9°56’E), with an extension of over 150 km^2^ (the maximum width is 11 km and the maximum length is 13 km) and an average depth of 7 m (Fig 1). It connects with the Mediterranean Sea through a straight channel that is about 6 km long, 300 m wide, and 12 m deep (Fig 1). Bizerte Lagoon is set in a basement of ante-Neogene rocks [31] that was eroded and experienced several sedimentary phases during the Miocene [32]. It communicates in the south with Ichkeul Lake via the Tinja stream. The principal streams, which are non-permanent watercourses, whose flows depend on rainfall that feeds the lagoon with freshwater are Tinja Channel and Rharek, Ben Hassine, and Guenniche streams (Fig 1).

The region is characterized by a sub-humid climate with 600 to 800 mm of annual rainfall [33]. The rainy period coincides with the winter, from November to April, with maximum precipitation in February (219.5 mm). Summer and the rest of the year are dry, without any freshwater input from neighboring streams [34], and stronger seawater influence [35]. The seasonal gradient of water salinity in the Bizerte Lagoon is relatively large, ranging, on average, from 20 in winter to 40 in summer [36] when the water column warming can produce stratification [35]. This lagoon has been exploited for fishing activities for several centuries and for mussel farming since 1964. The mussel *Mytilus galloprovincialis*, the European flat oyster *Ostrea edulis*, and the Japanese oyster *Crassostrea gigas* have been intensively cultured in this lagoon for decades, and natural beds for the European clam *Tapes* (*Ruditapes*) *decussatus* are still present in the lagoon.

About 163,000 inhabitants (2004 census) live around the lagoon, of which are mainly concentrated (≈70%) in the town of Bizerte [36]. The other important urban centers bordering the lagoon are Menzel Bourguiba, Menzel Abderrahman, and Menzel Jemil (Fig 1). The most important industrial activities are located in the vicinity of Bizerte (cement factory and metals treatment), Menzel Jemil (dye works and metallurgy), and Menzel Bourguiba (with a naval port, El Fouledh steelworks, a military arsenal, and metallurgy) [34]. Some other industries, such as an iron and steel plant, a cement factory, and a refinery, are established outside of these towns [37].

Human population growth around the lagoon caused an increase in the discharge of wastewater into the lagoon [38]. Recently, two main wastewater treatment (of Menzel Bourguiba and Bizerte) stations have been installed around the lagoon. Since the commissioning of sewage from the Bizerte (October 1997) and Menzel Bourguiba (January 1998) stations, wastewater discharge into the lagoon has almost halted. Furthermore, this lagoon has also been subject to intensive maritime traffic and indirectly to several pollutants coming from oil and steel factories. The construction of dams on Ichkeul Lake, which supplies the lagoon with freshwater, has strongly affected its natural equilibrium [37], as it caused a dropped of water input from 165 to only 20 million m^3^ per year [39]. Any human intervention in the Mediterranean Sea, Ichkeul Lake, and the catchment area, particularly in the Menzel Bourguiba industrial zone (construction of dams, break-waves, and industrial waste) might alter the distribution of the biogeochemical properties of this sensitive ecosystem and, consequently, modify its environmental quality [40].

## Materials and Methods

Ten stations were sampled in the lagoon on 22 March 2013 (Fig 1A). The sampling took place during a jellyfish bloom event (Fig 1B). Three successful replicates were taken from each station between a depth of 7 and 12 m using a box-corer, as were splits of the first surface centimeter of each station. The samples were analyzed for living benthic foraminifera, bacterial, and pigment contents, and several abiotic sedimentary variables, including grain size, carbonates, acid volatile sulfides (AVS), simultaneously sequential extracted metals (SEM), total organic carbon (TOC) and total nitrogen content (TN), mineralogy, and magnetic susceptibility as well δ^13^C data. Physicochemical parameters, such as temperature, salinity/conductivity, and oxygen content in water, and pH, Eh, and oxygen content in sediments, were measured with a multiparametric probe at each station. During the fieldwork, sediments for the benthic foraminiferal analysis were treated with a solution of Rose Bengal (2 g of Rose Bengal in 1,000 ml of alcohol) to differentiate living (Bengal rose-stained) from dead specimens [11]. The sediments for geochemical analysis were held in polyethylene flasks and kept at approximately -4°C prior to analysis.

### Textural, mineralogical, and magnetic susceptibility analyses

A laser microgranulometer (Mastersizer S instrument, Malvern Instruments, Malvern, UK) was used to determine the sedimentary particle sizes (in the range from 0.05–2,000 μm) of the sediments in each sample after the removal of organic matter (with hydrogen peroxide) and carbonates (with hydrochloric acid 70%). The mineralogical composition of the sediments was analyzed in the <63 μm and <2 μm (clay) fractions by X-ray diffraction (XRD) techniques, according to the methodology adopted by Martins et al. [41]. Magnetic susceptibility of the sediments was measured in the total sediment with a portable KT-9 Digital Magnetic Susceptibility Meter by taking 10 successive readings per sample and using the mean values to obtain a reliable result.

### Chemical analyses

The evaluation of calcium carbonate (CaCO_3_) content in the sediments was based on the volumetric analysis of carbon dioxide (CO_2_), which is liberated during treatment with a 4N hydrochloric acid (HCl) solution. The TOC and TN contents were analyzed using a Perkin Elmer (Waltham, MA, USA) PE 2400 CHN system.

The method for chlorophyll *a* (Chl *a*) and phaeopigment extraction was quite similar to that applied in a water sample, except that it was necessary to suspend the fraction of sediment directly in acetone 90%, and then to allow the extraction to proceed for 12 h at -20°C, followed by spin-drying and spectrometry measurements [42].

The SEM and AVS analyses of sediments were made using a cold acid purge-and-trap technique [43], [44] and using the methodology described by Oueslati et al. [45].The available concentrations of Cr, Ni, Zn, Pb, Cu, Fe, Mn, and Co, as well as the total concentrations of reactive metals (ΣSEM) were evaluated. It is possible to predict the heavy-metal bioavailability and the toxicity of the sediment according to the relationship ΣSEM/AVS (referred as SEM/AVS). Under suboxic to anoxic conditions, if SEM/AVS < 1, Metal-A (Available Concentrations or Reactive Metals) concentrations are controlled by solubility (Ksp) of their sulfides and there are weak metal concentrations dissolved and bioavailable in pore-water. On the contrary, if SEM/AVS > 1, metal concentrations become high, toxic and bioavailable in pore-water. It is inferred that no potentially metal bioavailability exists for benthic organisms when SEM/AVS < 1 [46], [47], [48], [49], [50].

### Bacterial analysis

For total coliform (TC), fecal enterococci (FE), total mesophilic counts (TMC), sulfate-reducing (SR) bacteria and Vibrionaceae (Vb) enumeration in the sediment, 10 g of sample was suspended in physiological water containing 3% NaCl (w/v). All counting methods were based on cultivation by a standard plate counting agar procedure [51]. Marine agar (Difco, Becton Dickinson, Franklin Lakes, NJ, USA) was used to count the total number of mesophilic bacteria, and the incubation temperature was maintained at 32°C for mesophilic population counting and all plates were incubated for 72 h before counting. For TC enumeration, ten-fold dilutions (100 μL) of the sediment samples suspension were spread on the surface of Desoxycholate Lactose agar plates. Counts were performed after incubation for 48 h at 37°C. Anaerobic SR bacteria were identified on the basis of their selective growth in Thio Sulfate Neomycin (TSN) agar medium (BioMérieux, Marcy l'Etoile, France) under an anaerobic atmosphere enriched to 5% CO_2_, according to the method of Larpent and Gourgand [52]. The estimation was realized after incubation for 72 h. For *Vibrionaceae* counting, the selective Thiosulfate-Citrate-Bile-Sodium agar (TCBS, Bio-Rad, Hercules, CA, USA) was used, and the total VB were counted after incubation for 72 h at 25°C [53].

### Foraminiferal assemblages analysis

On splits of the >63 μm sediment fraction, at least 300 living specimens were picked, counted, and identified. The relative abundance of each species was determined to characterize the living assemblages. Foraminiferal density (FD) and Shannon index (H’) [54] were analyzed according to the procedure adopted by Martins et al. [2]. The benthic foraminiferal diversity also was assessed through the species richness (S) and H’. The species equitability was evaluated through the equation J = H’ /lnS.

### Carbon isotopes

About 20 specimens of a living *Ammonia parkinsoniana* were collected from the sediment fraction 300–350 μm for carbon isotopic analysis on their tests. This taxon was selected since its present in all the studied stations. The sediment fraction 300–350 μm was used to avoid ontogenetic variations due to biological vital effects [55]. The "Kiel IV Carbonate Devise" equipment and "Delta V Plus—Isotope Ratio MS" analyzer (Thermo Scientific) were used for this analysis. The obtained results are compared with a standard carbonate, the Pee Dee Belemnite (PDB). Data from carbon isotopes are shown by the δ parameter defined by: δ (‰) = [R_sample_—R_standard_ /R_standard_) × 1000. In which, R values correspond to C_13_/C_12_. The accuracy of the analysis is 0.030 ‰.

### Data analysis

Selected logarithmically transformed variables [log (x+1)] were submitted to an R-mode and Q-mode cluster Analysis (CA) and principal components analysis (PCA). These analysis were conducted in Statistica 7.0 software. The R-mode CA was based on the “weighted pair-group average” algorithm for agglomeration, and the 1-Pearson r correlation, as a distance measure and the Q-mode CA was based on the Ward’s method and Euclidean distance.

The percentage of benthic foraminiferal species and some taxa/groups that reached a relative abundance ≥ 2% in at least one station and an occurrence in at least three stations were used in these analyses. Abiotic variables were selected according to their relevance to the aim of this work and, pattern of variability in the study area.

The station LB1, located in the channel connecting the lagoon with the sea was not considered in both analyses, because it had the lowest FD, diversity indexes and J and was characterized by an assemblage largely dominated by one species (*A*. *parkinsoniana*, reaching 91%) and it was therefore considered as an outlier.

## Results

### Abiotic Variables

**Fig 2:**
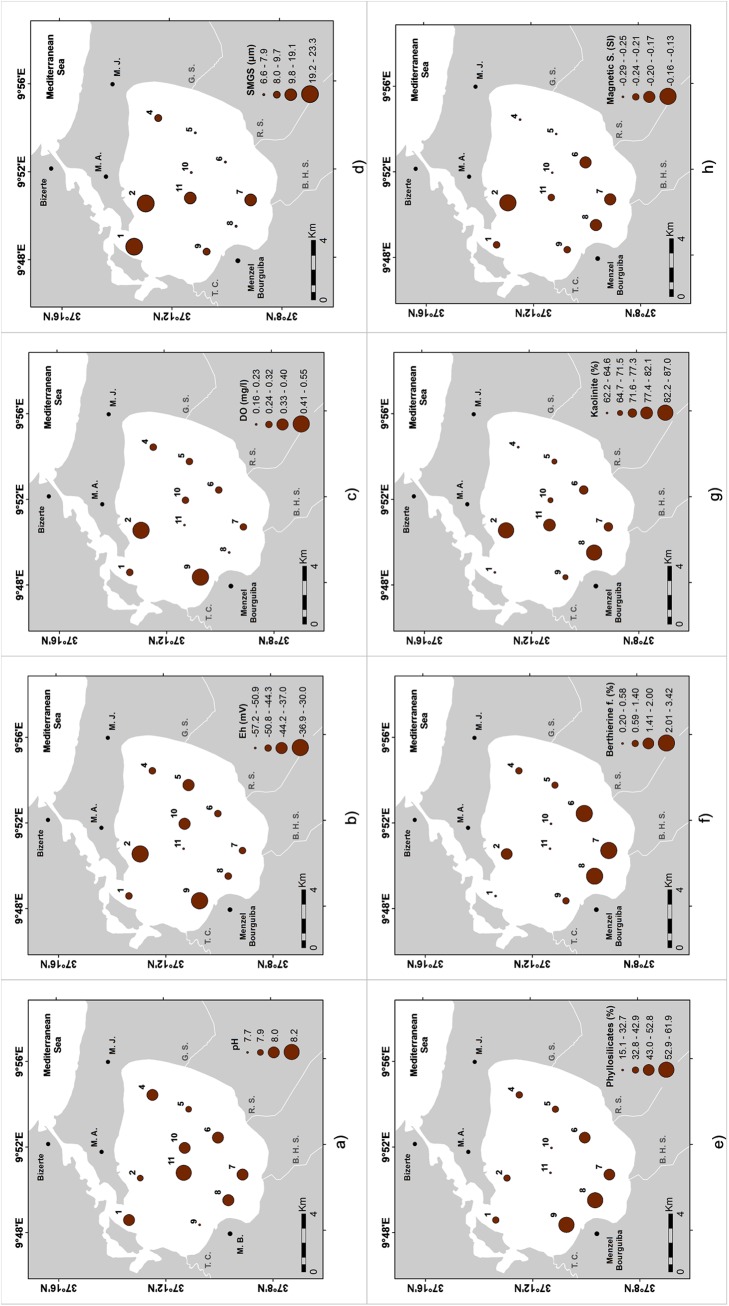
Distribution maps of: a) pH; b) redox potential (Eh) (mV); c) dissolved oxygen (DO) content in surface sediments (mg/l); d) sediment mean grain size (SMGS; μm); e) phyllosilicates (%); f) ferrous berthierine (f.; %); g) kaolinite (%); h) magnetic susceptibility (SI unities).

The sample locations, the abiotic variables, and the main biotic variables analyzed in this work are presented in S1 Appendix. The water temperature varied between 14.7 and 15.8°C, salinity from 28 to 33.3, pH ranged from 7.7 to 8.2 (Fig 2A) and Eh values from -57.2 to -30.0 mV measured in the sediment subsurface (Fig 2B). The DO in the water column (0.09–0.18 mg/l) was lower than in the surface sediment pore-water (0.16–0.55 mg/l; Fig 2C). The gray color of sediments several millimeters below the surface indicated reducing conditions agreeing with the negative Eh values. Relatively low values of Eh and DO values were also found in the inner part of the lagoon.

In the studied sites, the sediment mean grain size (SMGS) varied between 6.6–23.3 μm (Table 1), including mostly a fine gray muddy fraction (<63 μm; 76–100%). The sand fraction (>63 μm) was composed mostly of shell debris, which resulted in the textural characteristics of the sediments (bimodal to polymodal, poorly to very poorly sorted sediments). The coarsest sediment samples were found in the northern area near the connection with the Mediterranean Sea and at sites located near the streams’ mouths (Fig 2D).

**Table 1:** Selected sedimentological data.

Stations	SMGS	CaCO_3_	TOC	TN	C/N	Chl *a*	δ^13^C	AVS	SEM/AVS
LB-1	20	21	5.93	1.25	4.74	196.7	-1.78	212.23	0.52
LB-2	23.3	16	4.02	0.92	4.37	91.4	-1.17	617.28	0.16
LB-4	9.3	28	5.87	1.26	4.66	127.4	-2.32	242.33	0.37
LB-5	6.6	23	4.87	0.78	6.24	699	-2.21	181.32	0.54
LB-6	7.8	20	3.93	0.57	6.89	155.1	-2.4	65.96	2.6
LB-7	18.1	18	3.57	0.78	4.58	92.3	-1.64	0.0	
LB-8	6.6	12	2.54	0.88	2.89	244.7	-1.51	212.4	0.42
LB-9	7.9	18	3.29	2.25	1.46	69.3	-2.52	35.56	3.56
LB-10	7.7	21	4.02	1.09	3.69	241.9	-1.77	193.67	0.59
LB-11	9.7	19	3.77	1.05	3.59	122.8	-2.36	695.19	0.16
**Max.**	**23.3**	**28**	**5.9**	**2.3**	**6.9**	**699**	**-1.2**	**695**	**4**
**Min.**	**6.6**	**12**	**2.5**	**0.6**	**1.5**	**69.3**	**-2.5**	**0.0**	**0.2**
**Med.**	**11.7**	**19.6**	**4.2**	**1.1**	**4.3**	**204.1**	**-2**	**245.6**	**1**

SMGS–sediment mean grain size (μm); CaCO_3_ (%); TOC (%), TN—total nitrogen (%); C/N ratio; Chlorophyll a (Chl *a*; mg/m^3^); δ13C (‰; VPDB); AVS (μg g^-1^); SEM/AVS ratio.

Sediments were mostly composed of phyllosilicates (15–62%; mean 42%: Fig 2E), calcite (13–40%; mean 26%), quartz (9–60%; mean 18%), pyrite (0.2–15%; mean 3%), and anatase (<10%; mean 3%), along with minor proportions of K-feldspars (1–5%), plagioclase (<4%), anhydrite (<5%), ferrous berthierine (<4%: Fig 2F), dolomite (<2%), siderite (<2%), magnetite/maghemite (<1%), and hematite (<0.5%) (S1 Appendix). The assemblage of clay minerals was mainly represented by kaolinite (62–87%; mean 74%; Fig 2G), illite (2–35%; mean 21%), chlorite (<21%; mean 4%), and smectite (<2%). Sediments were more enriched, for instance, in phyllosilicates at the inner stations of the southern-western side of the lagoon (Fig 2E), calcite near the lagoon entrance and at stations of the eastern side of the lagoon, pyrite at some stations in the center of the lagoon, ferrous berthierine in the southern part of the lagoon (Fig 2F) and kaolinite at some stations of the external sector and central part of the lagoon (Fig 2G). The values of the sediments’ magnetic susceptibilities were negative at all the sites ranging from -0.29 to -0.13 SI unities (Fig 2H). The highest values were recorded in the northern and southern sides of the lagoon.

**Fig 3:**
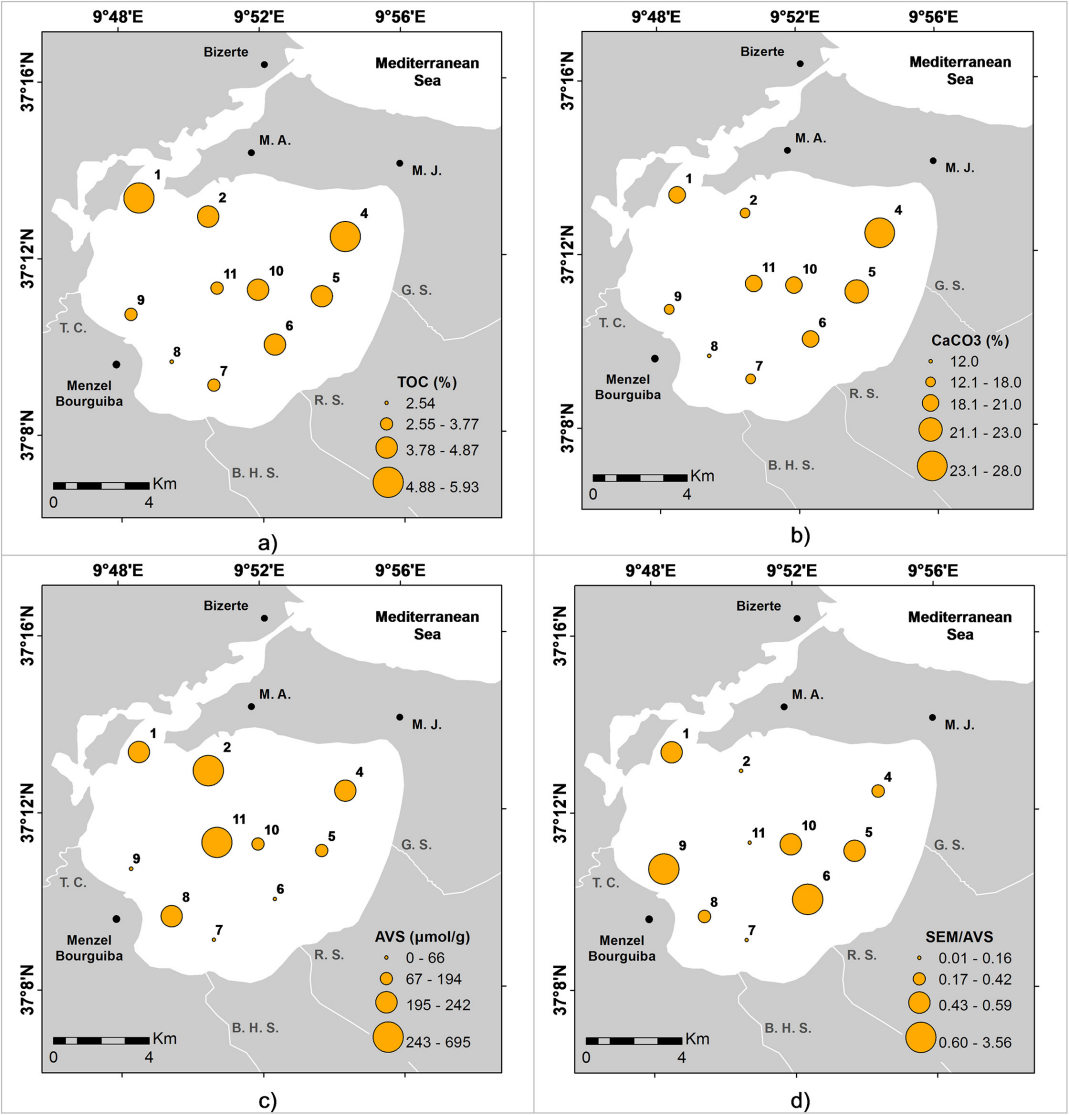
Distribution maps of: a) total organic carbon (TOC; %); b) calcium carbonate content (CaCO_3_; %); c) acid volatile sulfides concentrations (AVS; μg/g); d) simultaneous extracted metals and AVS ratio (SEM/AVS).

The CaCO_3_, TOC, TN, and the C/N ratio ranged from 12–28% (mean 20%), 2.5–5.9% (mean 4.2%), 0.6–2.3% (mean 1.1%), and 1.5–6.9 (mean 4.3), respectively (Table 1). The highest values of TOC (Fig 3A) and CaCO_3_ (Fig 3B) were found in the northern and eastern area of the Bizerte Lagoon. The AVS values and ΣSEM varied between 0 and 695 μg g^-1^ and 80 and 172 μg g^-1^, respectively (Table 1). AVS were found at all the stations (except LB7). The AVS values were higher in the northern and central areas of the Bizerte Lagoon (Fig 3C). The ranges of available concentrations of reactive metals were: Fe, 78–168 μg g^-1^; Mn, 0.9–2.22 μg g^-1^; Zn, 0.67–1.45 μg g^-1^; Cr, 0.09–0.24 μg g^-1^; Ni, 0.05–0.12 μg g^-1^; Pb, 0.04–0.11 μg g^-1^; Cu, 0.06–0. 1 μg g^-1^; and Co, 0.002–0.031 μg g^-1^ (S1 Appendix). Most of the accumulated reactive metals (ΣSEM) were higher in sites LB-1, LB-6 and LB-9 with concentrations of 110.1 μg g^-1^, 171.7 μg g^-1^ and 126.7 μg g^-1^, respectively. On the other hand, SEM/AVS > 1 values were found at sites LB-6 and LB-9 (Table 1). In this case, the trace metal concentrations in pore-water resulted high near the Tinja Channel and the Rharek Stream (Fig 3D).

**Fig 4:**
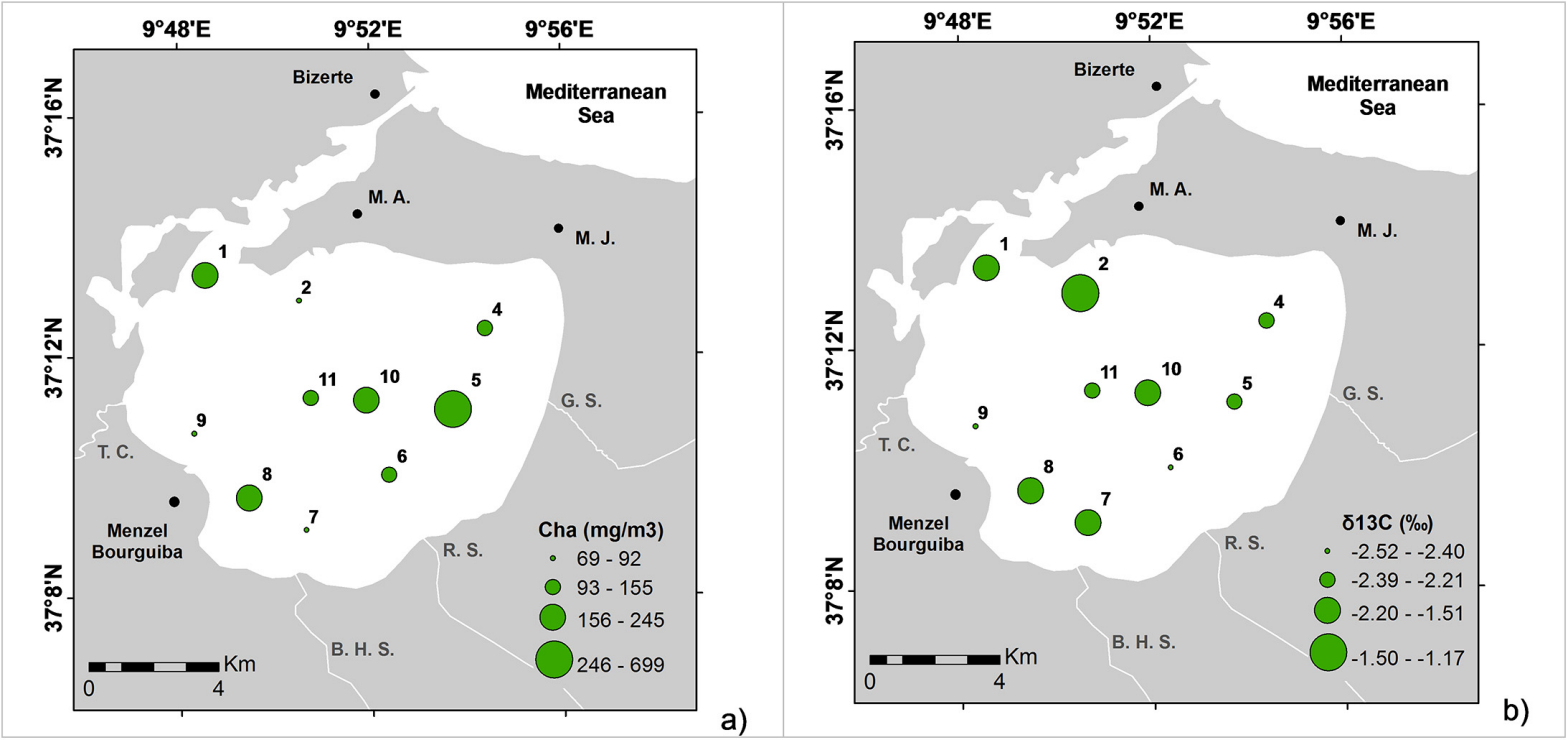
Distribution maps of: a) Cha—Chlorophyll a (Cha; mg/m^3^) and; b) δ^13^C (*‰*) values.

Chlorophyll *a* contents varied from 69–699 mg m^-3^ (mean 204 mg m^-3^; Table 1). Pheophytin concentration was below the detection limit. The highest concentrations of chlorophyll *a* were found in front of Guenniche Stream, in the channel connecting the lagoon with the Mediterranean Sea and near the Menzel Bourguiba town (Fig 4A). The *A*. *parkinsoniana* δ^13^C values oscillated between -1.2 and -2.5 ‰ (mean -2.0 ‰; Table 1). The highest δ^13^C values were recorded in the northern area of the lagoon whereas the lowest values were mostly found in stations located near the streams outflow and in the central part of the lagoon (Fig 4B).

### Biotic Variables

**Fig 5:**
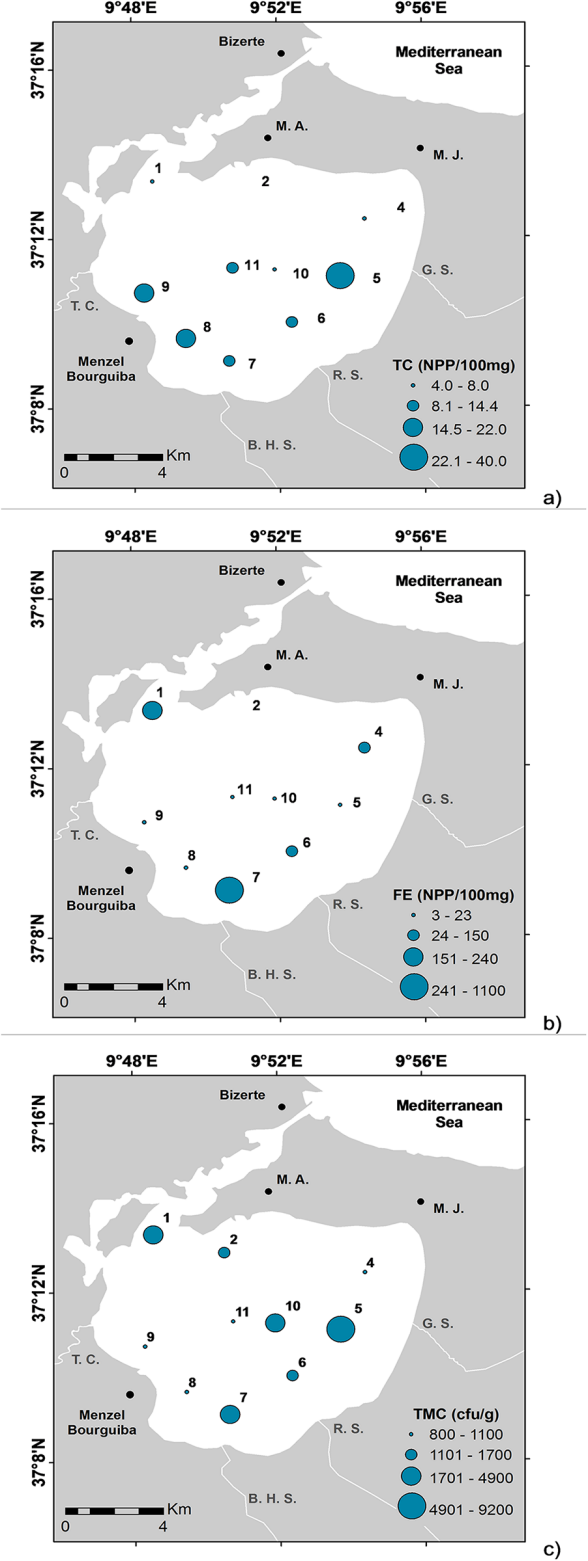
Distribution maps of bacterial counts: a) total coliforms (TC; net primary production per 100 mg or NPP/100 mg); b) fecal enterococci (FE; NPP/100 mg), and c) total mesophilic counts (TMC; colony-forming units per gram—cfu/g).

Bacterial counts (Table 2) revealed that the TC varied from 3–40 (NPP/100 mg: “Nombre le Plus Probable” or “Most Probable Number” per 100 mg), FE ranged from 3–1,000 (NPP/100 mg), and TMC, which includes all bacteria growing at temperatures ranging between 15°C and 45°C, was the most represented group, ranging from 8x10^2^ to 9x10^3^ (colony-forming units per gram, CFU g^-1^). SR bacteria were present in all samples, indicating the occurrence of anoxic conditions for almost all stations. The abundance of TC was higher in the inner part of the lagoon, in front of the main streams, indicating exogenous discharges (LB5, LB6, LB7, LB8, and LB9) (Fig 5A); FE reached locally high values (Fig 5B), and TMC increased in stations in both the outer and inner lagoonal zones (Fig 5C). TC abundance were higher than FE values, indicating an anthropogenic discharge at some stations.

**Table 2:** Selected biotic data.

Stations	S	J	H'	FD	TC	FE	TMC
LB-1	17	0.2	0.5	557	4	240	3400
LB-2	46	0.7	2.9	11096	<3	<3	1700
LB-4	35	0.6	2.3	2326	7	90	800
LB-5	28	0.6	1.9	3079	40	3	9200
LB-6	37	0.6	2.1	4113	14	150	1600
LB-7	39	0.5	1.9	5576	14	1100	4900
LB-8	33	0.7	2.3	3830	15	3	1100
LB-9	36	0.6	2.2	4299	15	21	1100
LB-10	38	0.7	2.4	14862	4	23	3000
LB-11	36	0.6	2.3	7132	9	20	1000
**Max.**	**46**	**0.7**	**2.9**	**14862**	**40**	**1100**	**9200**
**Min.**	**17**	**0.2**	**0.5**	**557**	**4**	**3**	**800**
**Med.**	**34.5**	**0.6**	**2.1**	**5686.9**	**13.6**	**183.3**	**2780**

S—species richness; J–equitability; H’—Shannon Index; FD—foraminifera density (n° g^-1^); TC—Total Coliforms; FE—Faecal Enterococci (NPP/100mg) and; TMC—Total Mesophyllic Counts (CFU g^-1^).

**Fig 6:**
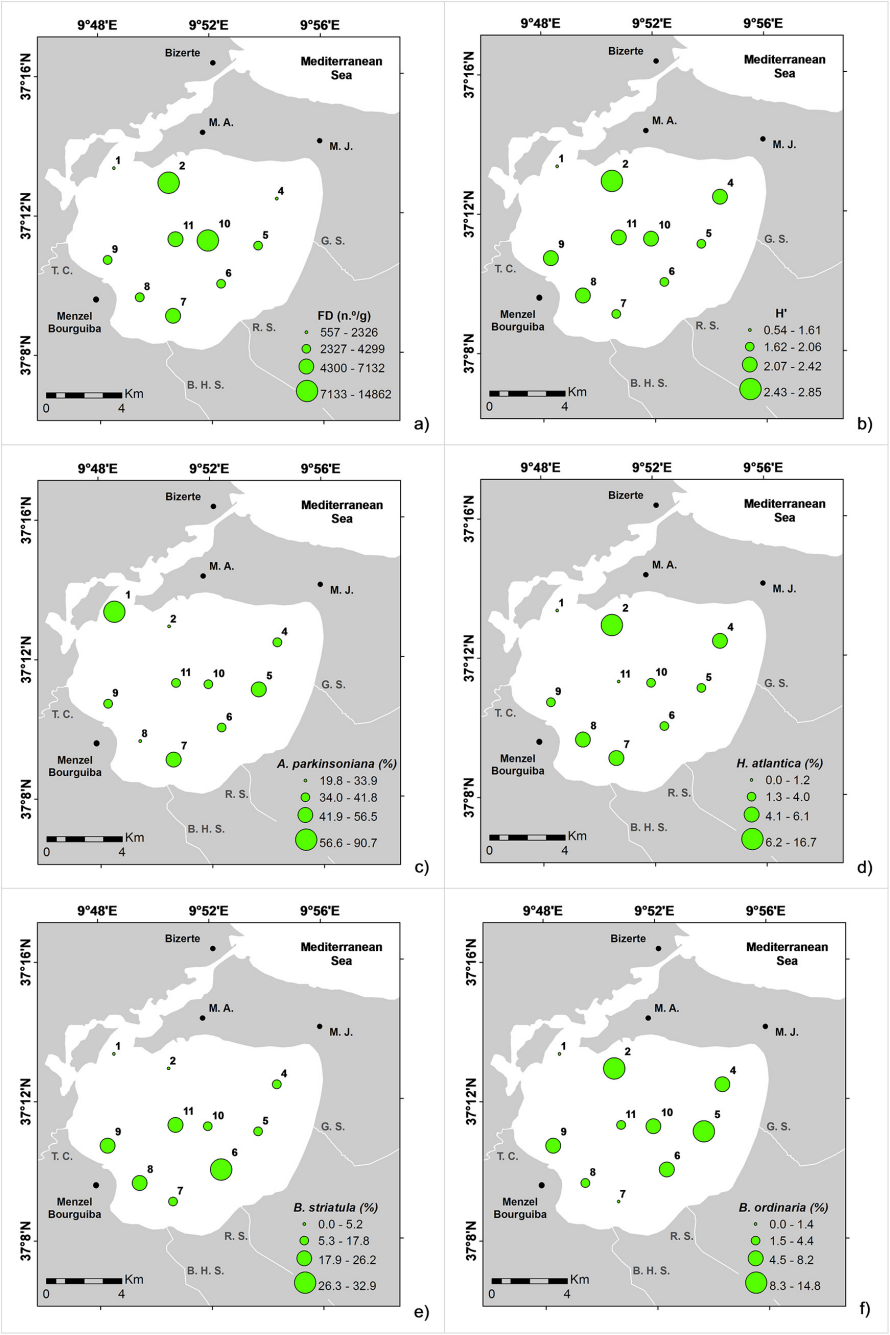
Distribution maps of: a) FD: foraminiferal density (n°/g); b) H’: Shannon Index; c) *Ammonia parkinsoniana* (%); d) *Hopkinsina atlantica* (%); e) *Bolivina striatula* (%); and; f) *Bolivina ordinaria* (%).

The FD ranged from ~557–14,862 n° g^-1^ (Table 2), and was particularly high at some stations in the northern and central parts of the lagoon (Fig 6A). A total of 87 species of living benthic foraminifera were identified (S2 Appendix). Species richness varied between 17 and 46, the H’ index ranged from 0.5 to 2.9 (Fig 6B), and J from 0.2 to 0.7 (Table 2). The lowest values of these variables were found at the station (LB1) located in the channel that connects the lagoon with the sea.

The foraminiferal assemblage of the Bizerte Lagoon was mainly represented by *A*. *parkinsoniana* (20–91%: dominant at all the sites), *Bolivina striatula* (up to 33%), *Hopkinsina atlantica* (up to 17%), and *Bolivina ordinaria* (up to 15%). Assemblages also included species such as *Elphidium gunteri* (<5%), *Bolivina compacta* (<4%), *Quinqueloculina seminula* (0.5–4%), *Bolivina pseudoplicata* (<4%), *Triloculina rotunda* (<4%), *Bulimina aculeata* (<4%), as well as those of minor relative abundance (<3%), for instance, *Ammonia tepida*, *Ammonia beccarii*, *Bolivina dilatata*, *Bolivina spathulata*, *Bolivina subaenariensis*, *Bulimina elongata/B*. *gibba*, *Bulimina marginata*, *Fursenkoina squamosa*, *Nonionella atlantica*, *Nonionella iridea*, *Spiroloculina excavata*, and *Triloculina trigonula*. *Ammonia parkinsoniana* (Fig 6C), *H*. *atlantica* (Fig 6D), and *Elphidium* spp. reached their highest relative abundances in the outer part of the lagoon. *Bolivina striatula* was mostly represented in the inner part of the lagoon (Fig 6E), and *B*. *ordinaria* was mostly found in the eastern side (Fig 6F). Bolivinids were quite common throughout the lagoon, while buliminids, as well as the agglutinated foraminifera, were prevalently found in the northern sector near the channel that connects the lagoon with the Mediterranean Sea.

### Results of statistical analyses

**Fig 7:**
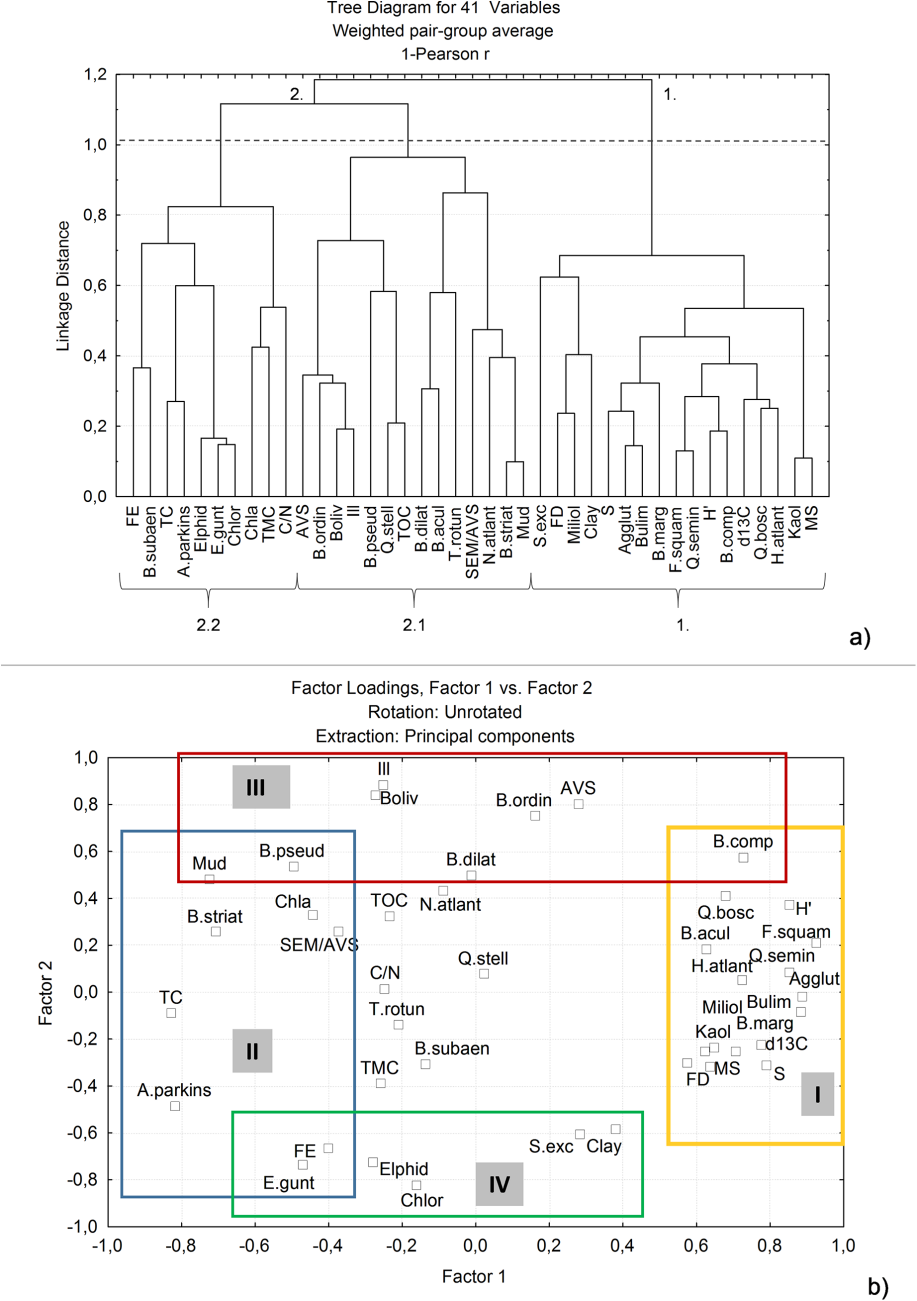
Principal components analysis based on the main benthic foraminifera species/taxa and: a) sedimentological data (grain size, mineralogical, geochemical and geophysical data); b) quantity and quality of organic matter and bacteria. Legend: Sand–sand fraction (>63μm); Mud–fine fraction (<63μm); clay–clay fraction (<2μm); total organic carbon–TOC; Chl–chlorite; Kaol–kaolinite; Berth–berthierine; Phyl–phyllosilicates; MS–magnetic susceptibility; AVS–acid volatile sulfides; SEM/AVS–simultaneous extracted metals and AVS ratio; FE—fecal enterococci; TC–total coliforms; TMC–total mesophilic counts; TBP—total of biopolymers; LIP–lipids; PTN–proteins; CHO–carbohydrates; Chla–Chlorophyll a; FD–foraminiferal density (n°/g); b) SI–Shannon Index; S–species richness; J–equitability.

The R-mode CA allows the identification of two main clusters of variables and two sub-clusters (Fig 7A). Cluster one includes foraminifera density and diversity indexes, δ^13^C values and several taxa/groups of foraminifera such as buliminids, miliolids and agglutinated species. Cluster 2 includes for instance *A*. *parkinsoniana*, bolivinids, elphidiids, bacteria, AVS and SEM/AVS values.

The PCA help us to clarify the relationship between the variables. It allows the identification of four groups of variables with similar patterns of distribution, considering the two first factors which explain together 53% of data variability (Fig 7B). The first factor is related to favourable/less favourable environmental conditions whereas the second one to the production of AVS and urban organic discharge. According to these two first factors, TOC content, C/N and TMC are secondary and less important variables. Numbers of taxa such as miliolids, including *Q*. *seminula* and *Quinqueloculina bosciana*, agglutinans, *B*. *marginata* and other buliminids, *F*. *squamosa*, *B*. *compacta* and *H*. *atlantica*, as well as FD and diversity indexes are positively related to δ^13^C, magnetic susceptibility and kaolinite content. Otherwise these variables are negatively related to SEM/AVS, TC and Chl *a*, which are instead grouped with *A*. *parkinsoniana*, *B*. *striatula* and *B*. *pseudoplicata*. The PCA grouped *B*. *ordinaria*, *B*. *dilatata*, *B*. *pseudoplicata*, *B*. *compacta* and other bolivinids with AVS, illite and mud content whereas *Elphidium* spp., *E*. *gunteri* and *S*. *excavata* are enclosed with FE, clay fraction and chlorite content.

**Fig 8:**
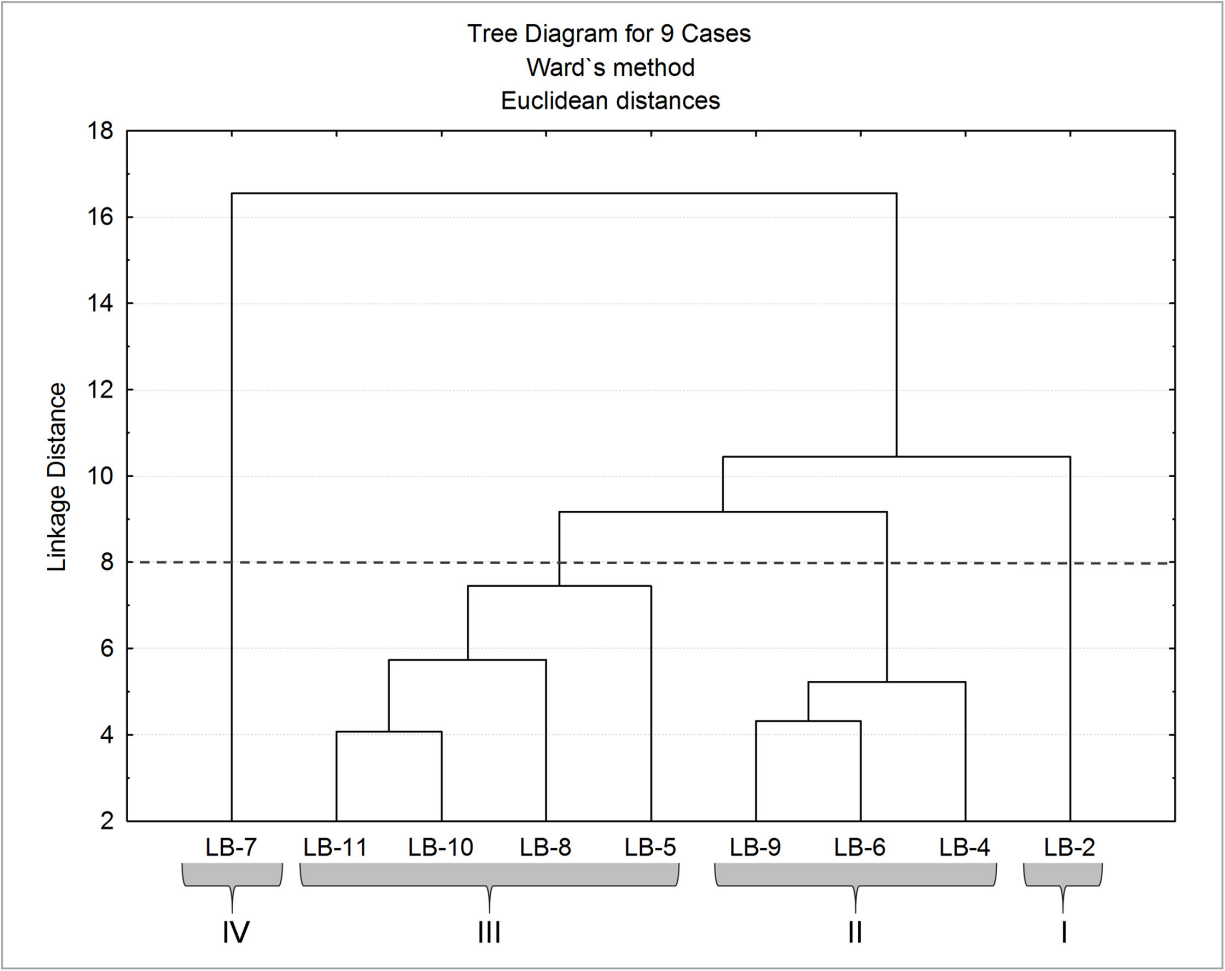
Results of Q-mode cluster analysis.

Q-mode cluster analysis recognized four groups and sub-groups of stations (Fig 8). Group 1 represented by only one station (LB2) located in the outer sector of Bizerte Lagoon, which is characterized by relatively coarse grain sediments, polymodal and very poorly sorted particles, with the highest TN, kaolinite, magnetic susceptibility and AVS values, and relatively high TOC content. High FD and the highest diversity indexes, J and δ^13^C values were found in this station as well as the highest percentages of *Bulimina* spp., *B*. *marginata*, *F*. *squamosa*, agglutinans, miliolids, *H*. *atlantica*, *Q*. *seminula* and *B*. *ordinaria*. Group 2 includes the stations LB4, located in front of Menzel Jemil, LB6, nearby the Rharek mouth and LB9 close to the Tinja Channel. These stations are marked by very fine sediments, composed tendentiously by bimodal and poorly sorted particles, and high available concentrations of Fe and ΣSEM and SEM/AVS values (except in LB4) and bolivinids percentage and relatively high values of TOC, TN, CaCO_3_, Chl *a*, phyllosilicates, illite and kaolinite. Foraminiferal density, magnetic susceptibility and δ^13^C have the lowest values in this group. Group 3, which includes the stations LB5, LB8, LB10 and LB11, located at the central area of the lagoon, is characterized by the finest sediments, composed tendentiously by bimodal and poorly sorted particles. In these stations, pyrite and Chl *a* reach the highest concentrations (except LB11) and the sediments have relatively high AVS values, TC, FD and bolivinids percentage. Group 4 is composed by the station 7, located in front of the Ben Hassine Stream. It has relatively coarse sediments, with polymodal and very poorly sorted particles. This station has the highest FE, TMC, and chlorite content and relatively high ferrous berthierine and C/N values as well as percentage of *A*. *parkinsoniana*, *Elphidium* spp. and *E*. *gunteri*.

## Discussion

### Possible causes of environmental stress for benthic organisms in Bizerte Lagoon

The presence of mostly fine-grained sediments at all of the studied sites indicates that the bottom currents are essentially dominated by a low hydrodynamic regime in most parts of the lagoon. However, relatively coarser sediments were found in the northern sector, near the channel connecting the lagoon to the sea, indicating a relatively dynamic current activity, as well as in the station in front of the Ben Hassine Stream, which is probably related to episodically coarser-grained contributions provided by this stream. The grain size data and the mineralogical composition of sediments show that the streams flowing into the lagoon are particularly enriched in fine detrital siliciclastic materials, such as phyllosilicates and clay minerals. Phyllosilicates were significantly higher in sediments near the Tinja Channel, Ben Hassine, and Rharek streams. The sediments located near these latter streams also had relatively high magnetic susceptibility values and ferrous berthierine and magnetite/maghemite contents. Nearby, the Ben Hassine Stream sediments were also relatively enriched in kaolinite, chlorite, and dolomite. The plagioclase content also increased in sediments close to the Rharek Stream.

Phyllosilicates are normally related to fine-grained sediments, and magnetic susceptibility depends on the abundance of magnetic minerals. In the studied stations, low amounts of magnetic minerals were identified by XRD that agree with the negative values of this variable. The lowest values of magnetic susceptibility were recorded in sediments enriched in organic matter and carbonates. The highest ferrous berthierine content was associated with the clay fraction, phyllosilicates (namely, kaolinite) that suggests that this mineral might have been introduced into the lagoon by streams. It was probably supplied by the erosion of the neighboring geological grounds of Jbels Kchabta and Messeftine (south of the lagoon) that would have delivered selected detrital materials to the lagoon [56].

However, the occurrence of ferrous berthierine materials associated with a high iron content and the clay fraction suggests that this mineral should be formed, at least in part, within the lagoon. According to Oliveira and Pereira [57], the berthierine ooids of the Devonian formations of Paraná Basin, in Brazil, have been deposited under a low sedimentation rate in shallow marine conditions interspersed by episodic transgressive events. This mineral can be formed in reductive, non-sulfide environments, where conditions may fluctuate from oxic to anoxic relatively easily [57]. Despite the formation of berthierine in recent sediments the process is as yet poorly understood. The distributions of the mineralogical composition of sediments, namely the phyllosilicates content as well as the magnetic susceptibility values, seem to result from both the differentiated sources of terrigenous materials and the action of the current surface lagoonal system. In addition, deposition of siliciclastic materials in some areas should eventually be a source of disturbance for benthic organisms.

The sediments have a relatively high calcite content in the northern (near the channel connection with the sea) and eastern areas of Bizerte Lagoon. Sources of calcite can be related in some areas to the mollusk cultivation in suspended structures (Fig 1C) that occurs in vast areas of the Bizerte Lagoon but is concentrated at the eastern part, delivering a high amount of carbonates to the lagoon bottom (Fig 3B). Abundant empty shells and living ostracods as well as foraminiferal tests were found at most stations, which may have also contributed to the relatively high values of calcite in the sediments. Carbonate concentration in these areas would prevent the decrease in pH during organic matter decomposition that is relatively important in the sediments of Bizerte Lagoon. Mollusk cultivation may further contribute to the increase in organic matter and fine-grained materials to the sediments, as suggested by TOC being increased in the same areas where this activity is particularly intense (Fig 3A).

A consequence of the aerobic degradation of organic matter is oxygen consumption and its decline in both sediments and in the water column. This effect is amplified by the long water-residence time that makes the Bizerte Lagoon highly sensitive to nutrient over-enrichment [58] and also to the blooms of organisms like cnidarian as observed during sampling (Fig 1B). Surface sediments in the studied sites were, however, oxygenated, as shown by DO values. This oxygenation was most probably promoted by the benthic microalgae that covered the lagoon bottom. The well-oxygenated surface sediments favored the development of benthic foraminiferal populations and other organisms such as the observed ostracods. On the other hand, the high amount of organic matter associated with fine-grained sediments favors oxygen depletion and establishment of sub-oxic and/or anoxic conditions in the sedimentary pore-waters below the surface, as indicated by the presence of pyrite, a mineral formed in anoxic environments [59] and by the reduced values of redox potential. Pyrite is formed in neutral and alkaline solutions, like those found in the sediments of Bizerte Lagoon, and is preserved in anoxic layers [60]. The presence of AVS at all stations (except in LB2) is indicative of sulfide release that may harm benthic fauna.

Another source of stress for benthic organisms in Bizerte Lagoon is the presence of contaminants such as metals. Metal bioavailability in sediments has been discussed by several authors [61], [62], [63], [64], [65]. The ratio SEM/AVS > 1 in the southern part of the lagoon (LB-6 and LB-9), due to the water inputs in this area from Tinja Channel and Rharek Stream. In both areas, trace metal concentrations become relatively high in pore water and can eventually affect benthic foraminifera.

The PCA results indicate that an SEM/AVS increase signals a reduction in benthic foraminiferal abundance and diversity. It also suggests that the most tolerant species to an SEM/AVS increase might be *A*. *parkinsoniana*, *Bolivina striatula*, and *B*. *pseudoplicata*. Furthermore, the most tolerant species to sulphidic environments are *Bolivina* spp., namely *B*. *ordinaria*, *B*. *dilatata*, *B*. *pseudoplicata*.

### Organic matter and bacteria availability for benthic foraminifera

The C/N ratio (varying between 1.5 and 6.9; mean 4.3) indicates that the organic matter deposited on the sediments of the studied stations is provided by the lagoon’s biological productivity. Algae typically have C/N ratios between 4 and 10, whereas vascular land plants have C/N ratios of 20 or greater [66], [67], [68]. This difference is due to the presence of cellulose in vascular plants and the consequent relative enrichment of proteinaceous materials in the algae and animals living in this lagoon. In some stations (LB8-LB11) near the area influenced by the Tinja Channel, values of C/N < 4 were recorded, indicating a higher contribution of vascular plants.

Chlorophyll *a* concentrations are relatively high in the studied area, supporting the idea of a high benthic primary productivity or a high supply of fresh algal products at all stations, except at LB2, near the channel connecting the lagoon with the sea, and at LB9 and LB7, located nearby the Tinja Channel and the Ben Hassine Stream, respectively. The stations with low chlorophyll *a* concentrations also exhbit low TOC contents, probably due to the supply of siliciclastic materials by streams and currents.

The highest δ^13^C values were recorded in the outer and southern sectors of the lagoon (near the Ben Hassine Stream). Foraminiferal δ^13^C can be related to several parameters, such as respiration, CaCO_3_ precipitation rate, and the global carbon cycle, due to the partitioning of carbon between the terrestrial biosphere atmosphere and ocean reservoirs [69]. The living vegetation and soil organic matter have low δ^13^C values compared with that produced in the ocean [70]. In seawater, the δ^13^C values can be influenced by local changes in photosynthesis or respiration. Where photosynthesis dominates, the δ^13^C is relatively high, and it is relatively low where respiration predominates [69]. However, there is a consensus that carbon isotopes in foraminiferal carbonate tests are related to paleoproductivity and/or water mass properties [71], [72], [73], [74]. Results of this work given by both CA and PCA support a positive relationship between the δ^13^C values and FD, S, H’, and J, and several species/taxa (*Bulimina* spp., *B*. *marginata*, *F*. *squamosa*, agglutinans, miliolids, *H*. *atlantica*, *Q*. *seminula* and *B*. *ordinaria*) which agree to some extent with the above.

The highest abundance of total coliforms (TC), fecal enterococci (FE), and total mesophilic counts (TMC) were found mostly at stations related to the outflow of Tinja Channel and the Ben Hassine, Rharek, and Guenniche Streams, principally in areas related to the sources of inland waters surging to the lagoon. The increase of TC and FE is associated with a reduction in FD, S, H’, and J, as indicated by CA and PCA (Fig 7A and 7B). Only some species such as *A*. *parkinsoniana*, *B*. *striatula* B. *pseudoplicata*, and *E*. *gunteri* seem to respond positively to increases of FE and TC. These species also seem to have a relatively high tolerance to metal toxicity (according to CA and PCA results: Fig 7A and 7B). Otherwise, some bolivinids, such as *B*. *ordinaria*, *B*. *dilatata*, *B*. *pseudoplicata*, and *B*. *compacta* seem to be the most tolerant species to release of AVS.

### Assessing the sediment quality of Bizerte Lagoon

The Bizerte Lagoon foraminiferal assemblages are dominated by *A*. *parkinsoniana* and, locally, by *B*. *striatula*, *H*. *atlantica*, and *B*. *ordinaria*, and are composed by quite different species compared to those found in other Mediterranean transitional environments, as described by [7], [75], [76], [77], [78]. In the Lagoon of Venice (on the northern coast of the Adriatic Sea, Italy) [75], Santa Gilla Lagoon (Cagliari, Italy) [7], Orbetello Lagoon (Tuscany, Italy, on the coast of the Tyrrhenian Sea) [76], Lesina Lagoon (southern Italy, on the coast of the Adriatic Sea) [76] and Lake Varano (southern Italy) [77] *A*. *tepida* and *H*. *germanica* are the dominant species in the most confined areas, where the pressure caused by metal accumulation is high and brackish conditions occur. In the Ria de Aveiro, a transition North Atlantic ecosystem (Iberian Peninsula, Portugal), contaminated by metals, living assemblages of benthic foraminifera at the most polluted sites are also dominated by *A*. *tepida* and *H*. *germanica* [2], [8], [22], indicating a degree of environmental stress similar to that of referenced Mediterranean lagoons contaminated by several pollutants, including metals, as well as by agricultural and domestic effluents.

According to Coccioni et al. [75], *A*. *tepida* and *H*. *germanica* might be able to tolerate highly polluted environments and high concentrations of metals, whereas *A*. *parkinsoniana*, the principal species found in the Bizerte Lagoon, appears to be more sensitive to high metal levels. Metals and stressful conditions can inhibit metabolism and protein synthesis [79], causing a reduction in the diversity of foraminiferal populations.

The most impacted sites are stations LB6 and LB9 (group 2 of Q-mode CA; Fig 8), located nearby the Rharek Stream and the Tinja Channel, respectively, where high bioavailable concentrations of metals were found. These streams are possibly sources of metals to the lagoon and contribute to the reduction of foraminiferal abundance and diversity, as suggested by the results of PCA (Fig 7A). Station LB4 is part of this group but does not exhibit metal concentrations as high as the other stations, though it is characterized by a quite high TOC content and FE, and low δ^13^C, similar to LB6 and LB9, which suggests that urban discharges of organic materials in the lagoon and metals availability are having a negative impact on benthic foraminifera.

The stations located at the central area of the lagoon (LB5, LB8, LB10, and LB11, Group 3 of Q-mode CA; Fig 8) are dominated by *A*. *parkinsoniana* and bolivinids, namely by *B*. *striatula*. Benthic foraminiferal assemblages of this area are abundant and well-diversified as they might have benefitted from quite stable conditions at the bottom, with relatively low impacts caused by pollution and a high supply of organic matter. The highest abundance of bacteria was found in Station 7 (Group 4 of Q-mode CA; Fig 8), mostly FE and TCM. Foraminiferal assemblage was relatively lowly diversified, and dominated by *A*. *parkinsoniana*. They also containing a significant abundance of *E*. *gunteri* and other elphidiids and bolivinids. These characteristics might suggest that this is a relatively impacted area due to the supply of detrital materials (according to the textural and mineralogical parameters) and municipal pollution introduced in the lagoon by the Ben Hassine Stream.

The highest foraminiferal density, diversity, and equitability were found in the outer sector of Bizerte Lagoon (station LB2; Group 1 of Q-mode CA; Fig 8). This seems to be the least polluted area of the lagoon despite being affected by higher levels of AVS. The low level of confinement may reduce the negative effect caused by the release of sulphides, which is recognized to be harmful to benthic organisms [80]. Foraminifera are tolerant to short-term exposure to sulphide release and they can withstand these conditions but do not reproduce [81]. However, we do not know how long the AVS release has been taking place in this zone.

According to our interpretation, the variables that seem to contribute the most to foraminiferal distribution in Bizerte Lagoon are the sediments’ grain size and composition, as a result of stream runoff, currents and biogeochemical processes, organic matter quality, bacterial type, and abundance. According to Armynot du Châtelet et al. [82], these are important parameters driving benthic foraminiferal distribution. The concentrations of metals are reaching relatively high levels in some areas of Bizerte Lagoon and are beginning to exert a negative impact on benthic organisms. However, the contamination caused by metals is not yet of particular concern in this lagoon, as indicated by the relatively abundance of foraminifera and other benthic organisms such as ostracods, and the presence of typically marine species, such as *B*. *striatula*, *B*. *dilatata*, *B*. *subaenariensis*, *H*. *atlantica*, *B*. *aculeata*, *B*. *marginata*, *F*. *squamosa*, and *N*. *atlantica* [83] which are not commonly found in internal areas of Mediterranean coastal lagoons [7], [75], [76], [77], [78]. Data from this work suggests these marine species are entering and populating the Bizerte Lagoon due to favorable environmental conditions: relatively low water temperature, relatively high salinity, high insolation, low turbidity, good oxygenation on the superficial sediment, abundance of food, a saturated carbonate environment favorable to organisms with carbonated tests, and low disturbance of bottom sediments by currents.

## Conclusion

The mineralogical and geochemical compositions of sediments trace different pathways of sediment contribution by streams and by municipal, domestic, and industrial effluents into the Bizerte Lagoon. Once introduced, sediments are redistributed according to the lagoon’s hydrodynamics. The streams’ outflow introduces in some areas organic matter, metals, and bacteria. The availability of metals attains high levels at some stations in which signs of benthic foraminifera decline are plausible (reduction of assemblages’ dimensions and a decline in diversity). However, atypical benthic foraminiferal assemblages were found in Bizerte Lagoon, types not commonly found in contaminated transitional environments. These assemblages include several species common in continental shelf environments, in areas where the flux of organic matter is high. These species seem to be attracted by favorable environmental conditions and by the abundance of food. The composition of benthic foraminiferal assemblages found in Bizerte Lagoon allow us to suggest that this environment has not yet attained a degree of contamination by metals and organic matter of concern for benthic fauna.

## Supporting Information

S1 Appendix The abiotic and main biotic variables analyzed in this work. (XLSX)

S2 Appendix Percentage of living foraminifera species found in each studied station. (XLSX)
